# Learned Prediction of Compressive Strength of GGBFS Concrete Using Hybrid Artificial Neural Network Models

**DOI:** 10.3390/ma12223708

**Published:** 2019-11-10

**Authors:** In-Ji Han, Tian-Feng Yuan, Jin-Young Lee, Young-Soo Yoon, Joong-Hoon Kim

**Affiliations:** 1 School of Civil, Environmental and Architectural Engineering, Korea University, Seoul 02841, Korea; injihan@korea.ac.kr (I.-J.H.); yuantianfeng@korea.ac.kr (T.-F.Y.); 2 School of Agricultural Civil & Bio-Industrial Engineering, Kyungpook National University, Daegu 41566, Korea; jinyounglee@knu.ac.kr

**Keywords:** ground granulated blast furnace slag concrete, artificial neural network, particle swarm optimization, back-propagation, hybrid PSO-BP

## Abstract

A new hybrid intelligent model was developed for estimating the compressive strength (CS) of ground granulated blast furnace slag (GGBFS) concrete, and the synergistic benefits of the hybrid algorithm as compared with a single algorithm were verified. While using the collected 269 data from previous experimental studies, artificial neural network (ANN) models with three different learning algorithms namely back-propagation (BP), particle swarm optimization (PSO), and new hybrid PSO-BP algorithms, were constructed and the performance of the models was evaluated with regard to the prediction accuracy, efficiency, and stability through a threefold procedure. It was found that the PSO-BP neural network model was superior to the simple ANNs that were trained by a single algorithm and it is suitable for predicting the CS of GGBFS concrete.

## 1. Introduction

Numerous researchers have attempted to enhance the sustainability of concrete by not only reducing the amount of carbon dioxide (CO_2_) generated from the production of the Portland cement, but also increasing the durability of concrete, which can benefit the environment through the conservation of resources and the reduction of waste [1]. One commonly used strategy is to utilize recycled aggregates and mineral admixtures, such as fly ash, ground granulated blast furnace slag (GGBFS), and silica fume as a partial replacement for cement or aggregate in concrete [1,2,3]. The use of such industrial by-products has been found to improve the mechanical properties and durability of concrete, reducing CO_2_ emissions, conserving energy, and mitigating the adverse environmental effects of concrete [4]. 

Blast furnace slag is a by-product that was obtained in the production of iron in a blast furnace. When the molten blast furnace slag is quenched with water and finely ground to a cement parcel size, it is transformed into GGBFS. GGBFS, as a latent hydraulic material, reacts with calcium hydroxide (Ca(OH)_2_) in the presence of water, forming calcium silicate hydrate (C-S-H), which is primarily responsible for the strength of cement-based materials [5,6]. Through this pozzolanic reaction, the use of GGBFS as a supplementary cementitious material might reduce the early strength, but it increases the ultimate strength and significantly improves the microstructure and durability of hardened concrete [7,8,9]. 

Several empirical equations and mathematical models have been developed for estimating the compressive strength (CS) and other properties to minimize the experimental task that is required for concrete mix design [10,11,12]. These equations are generally in regression form based on the results of a series of experiments. However, selecting a suitable regression equation (linear, nonlinear, exponential, etc.) for each analysis requires considerable experience and multiple techniques, and the accuracy of analysis decreases as the number of explanatory variables increases [13,14,15]. In recent years, numerical modeling for such relationships has been accomplished by constructing an artificial neural network (ANN) model, which is capable of learning and generalizing from examples through the trial-and-error method without any presumptions [13,16]. ANNs can not only produce correct or nearly correct solutions to incomplete tasks, but also generate evidential results, even when the data are poor or insufficient [17,18]. Owing to these advantages, numerous researchers have applied ANNs for predicting the CS and other properties of concrete [4,19,20,21]. Bilim (2009) [21] used ANN models that were trained by several different back-propagation (BP) algorithms to predict the CS of GGBFS concrete based on concrete ingredients and age. Bakhta Boukhatem et al. (2011) [22] investigated the efficiency factor of GGBFS related with concrete strength by using ANNs.

In most studies employing ANN models for the estimation of concrete properties, a BP algorithm was used to train the network [19,20,21]. Nevertheless, the BP algorithm has some disadvantages: it can be easily trapped in local minima depending on the selection of initial parameters and it may be unreliable (with a low prediction accuracy), relying on training data [23,24]. Combinations of BP and several metaheuristic algorithms have been proposed as alternatives to overcome these drawbacks. Among the metaheuristic algorithms, particle swarm optimization (PSO) has been often integrated with BP algorithm to improve the performance of predictive models due to its simplicity and wide applicability. The hybrid PSO-BP algorithm uses the global search ability of PSO algorithm and the fast-converging capabilities of BP algorithm so that the ANN models with it can converge to true global optimization more accurately and rapidly than the models with a single algorithm. The effectiveness and superiority of this hybrid algorithm have been proven in various fields [25,26,27,28]. Bo et al. (2017) [27] proposed a hybrid PSO-BP neural network for wind power forecasting, and its performance was compared to the network that was trained by the conventional BP algorithm. The results of their study showed that the performance prediction of the developed hybrid algorithm is superior to the basic BP algorithm. Wang et al. (2015) [28] used the PSO-BP neural network to enhance the performance of the integrated navigation system and indicated that neural networks with the hybrid PSO-BP algorithm can compensate and estimate the navigation error more effectively than the conventional neural networks. However, few studies have been performed on the use of the hybrid algorithms to develop ANN models for predicting concrete properties. 

In this study, three different ANN models using BP, PSO, and hybrid PSO-BP algorithms were developed for predicting the CS of GGBFS concrete based on the concrete mix ingredients and curing temperature. The prediction results of these models were compared to investigate the beneficial effects of combining the BP and PSO algorithms and select the best intelligent system for the estimation of GGBFS concrete strength.

## 2. Database 

It is necessary to prepare data and construct a database for training and testing the prediction model to develop ANN-based models for predicting the CS of GGBFS-incorporated concrete. The 269 experimental data that were used in this study were collected from several reports [11,29,30,31,32,33,34,35,36,37]. All of the data contained complete sets of information regarding the mix design proportion, curing condition, and experimental CS of GGBFS concrete. The variables were selected according to all of the available data samples. The input parameters included the curing temperature (T), water to binder ratio (w/b), GGBFS to total binder ratio (GGBFS/B), water (W), fine aggregate (FA), coarse aggregate (CA), and superplasticizer (SP). The output variable was the CS at 28 days, which ranged from 17 to 80 MPa. Details regarding the chemical and mechanical properties of the concrete components are presented in [11,29,30,31,32,33,34,35,36,37]. Table 1 presents the minimum and maximum values of each parameter, and Appendix A presents a database containing all of the data. 

## 3. Methodology

### 3.1. Artificial Neural Network

**Figure 1:**
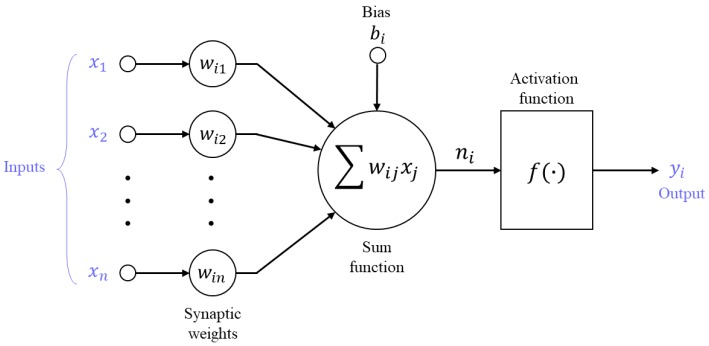
Artificial neuron model.

ANNs are massive parallel systems that are composed of simple, highly interconnected processing units, i.e., artificial neurons, which process information. ANNs are effective for engineering applications and they have been widely used to solve diverse problems due to its ability learning from examples [16,38]. ANNs can be classified into different types depending on the architecture and information flow procedure [15]. Among them, the multilayer feedforward network consisting of an input layer, one or more hidden layer(s), and an output layer is the most commonly used network, where all of the neurons in each layer only have connections to the neurons of successive layers, not to neurons in the same layer [15,17]. Every node in a layer is connected to the nodes in the adjacent layers with different weights. The typical elements of a neuron are shown in Figure 1: inputs, a summation function, an activation function, a bias, and an output. In every neuron except for the input neurons, signals from the previous layer (*x_i_*) are multiplied by an associated adaptive weight (*w_ij_*), which indicates the connection strength of the neuron with a particular input, and the summation function is then applied to the weighted signals [39]. Finally, the bias of the neuron (*b_j_*) is added to the aggregate signals, which forms the net input of the neurons (*n_i_*). This process can be mathematically expressed as:

(1) $$n_{i}=\overset{}{\underset{}{\sum }}w_{ij}x_{j}+b_{i}.$$

The output (*y_i_*) of the neuron is then obtained by applying an activation function (*f*) to the net input (*n_i_*):

(2) $$y_{i}=f\left(n_{i}\right).$$

The activation function limits the amplitude of the output of a neuron within a manageable range and introduces nonlinear properties to the neuron. In general, the hyperbolic tangent function is a commonly used activation function in multilayer models [15].

Training ANNs is a process of updating the connection weights and biases, so that the network exhibits desired or interesting behavior. In the course of training, the network architecture and parameters are adjusted by the iterative simulation with the given training examples to minimize the error function, which is often represented as the root mean squared error (RMSE), and to produce outputs that are equal or close to the targets [39,40]. Instead of following a set of rules that are specified by experts, ANNs automatically learn underlying rules from the given examples [41]. The steps used for training the network are called the learning algorithm. 

### 3.2. Back-Propagation

The BP algorithm is the most widely used algorithm for training ANNs [42]. It is a gradient-based procedure to minimize the error between the network outputs and the desired outputs, adjusting the weights and biases by a small amount at a time [15,17]. It comprises two procedures: a forward stage and a backward stage. In the forward procedure, the input signals move forward through the network and the error is calculated in the output layer. Subsequently, the error is propagated backward from the output layer to the input layer, updating parameters for the direction in which the performance function most rapidly decreases [40,42]. The change of the weights during each iteration is calculated, as follows:

(3) $$\Delta w_{k}=\alpha \Delta w_{k-1}-\eta \frac{\partial E}{\partial w},$$

where w is the weight, $\Delta w_{k}$ and $\Delta w_{k-1}$ are the changes in the weight w at *k* and *k*−1 iteration, α is the momentum factor, and η is the learning rate. The entire procedure is repeated until the performance of the network reaches an acceptable level.

### 3.3. Particle Swarm Optimization 

PSO is a stochastic optimization technique for finding the best solution, which is inspired by the social behavior of biological organisms to locate desirable positions in a given area through cooperation and competition [43,44,45,46]. In PSO, some entities, called particles, are scattered in the search space, and the position of each particle represents a possible solution to the optimization problem in the n-dimensional search space [46,47]. Each particle moves iteratively through the problem space to find the optimal locations, while remembering the best position it has ever visited and communicating with other particles. 

The position and velocity of the particles are randomly initialized at the beginning of the process and, during every iteration, each particle accelerates toward its own personal best solution discovered so far, as well as the global best position found thus far across the whole population [48]. The velocity and position of each particle are updated via the following equations at every step *t* [49]:

(4) $${\vec{v}}_{t+1}=w\times {\vec{v}}_{t}+c_{1}\times r_{1}\times \left(\vec{pbest}-{\vec{x}}_{t}\right)+c_{2}\times r_{2}\times \left(\vec{gbest}-{\vec{x}}_{t}\right),$$

(5) $${\vec{x}}_{t+1}={\vec{x}}_{t}+{\vec{v}}_{t+1},$$

where ${\vec{v}}_{t+1}$, ${\vec{v}}_{t}$, ${\vec{x}}_{t+1}$, and ${\vec{x}}_{t}$ represent the new velocity, current velocity, new position, and current position of the particles. $r_{1}$ and $r_{2}$ are random numbers uniformly distributed in the range of (0, 1) [50], giving the particles good state space exploration ability. $c_{1}$ and $c_{2}$ are referred to as acceleration coefficients, which represent the strength of attraction toward the personal best position ($\vec{pbest}$) and the global best position ($\vec{gbest}$), respectively [50,51]. The velocity (${\vec{v}}_{t})$ is updated based on its current values multiplied by the inertia weight and the distances from its current position to the personal best and the global best. The particle position (${\vec{x}}_{t})$ is adjusted according to the newly computed velocity (${\vec{v}}_{t+1}$). Subsequently, the fitness of each updated position is evaluated, and the personal best and global best are updated during each iteration. This process is repeated until the expected position is obtained or the termination criteria are satisfied. 

### 3.4. Hybrid PSO-BP Algorithm

The hybrid PSO-BP algorithm that is proposed herein is an optimization method that combines the PSO with the BP. Although the BP algorithm is the most widely used training algorithm for ANNs, it can easily fall into the local optimal solution, and its performance depends on the initial weights of the ANN [23,27]. If the initial weights and biases are far from the optimal values that can give the global optimal solutions, the ANN might become stuck at the local minimum [23]. Many researchers have combined the BP algorithm with metaheuristic optimization algorithms, such as PSO, genetic algorithm, and harmony search algorithm, to overcome these shortcomings of the BP algorithm and enhance the accuracy of models [23,45,52]. Among them, PSO has been often used to improve the performance of BP training in ANNs due to its simplicity and wide applicability [27,52]. 

**Figure 2:**
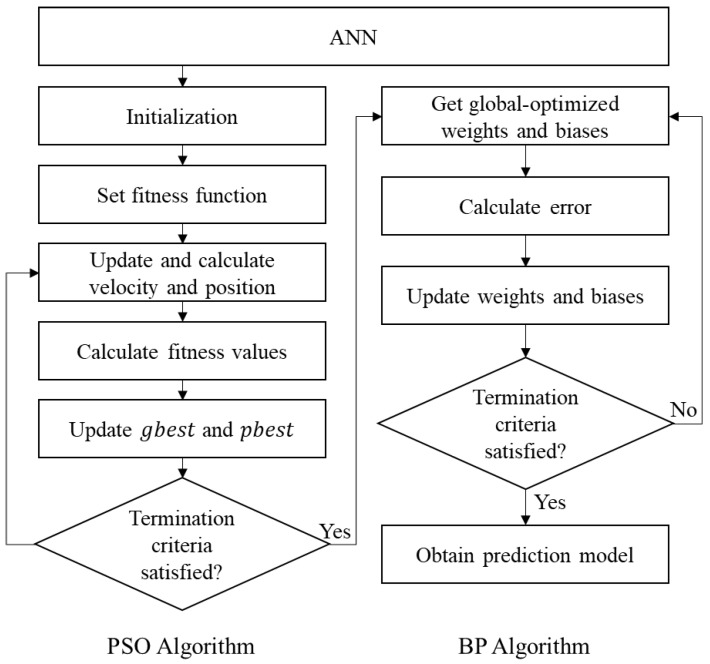
Flowchart for the hybrid particle swarm optimization-back-propagation (PSO-BP) algorithm.

The hybrid PSO-BP algorithm employs the global search ability of the PSO algorithm to obtain the initial weights and biases of the ANN that can lead the network to converge to the global minimum of the error function, and it uses the fast-converging capabilities of the BP algorithm. The near-global optimal initial weights and biases that were obtained by the PSO algorithm were applied in BP training to find true global optimization and improve performance of the ANN. Figure 2 describes the overall calculation process of the PSO-BP algorithm that was used in this study. Section 4 provides details regarding the determination of the parameters and the modeling of the PSO-BP ANN for predicting the CS of concrete.

## 4. Development of CS Prediction Models 

This section presents the procedures for developing the ANN models while using the BP, PSO, and PSO-BP algorithms for predicting the CS of GGBFS concrete. As previously mentioned, the curing temperature (T), water to binder ratio (w/b), GGBFS to total binder ratio (GGBFS/B), water (W), fine aggregate (FA), coarse aggregate (CA), and superplasticizer (SP) were used as the input parameters for the CS prediction models. To construct and evaluate the network models, the dataset was divided into training and testing sets; 80% of the data were used for training and the remaining 20% were employed for testing. For the BP algorithm, 10% of the training dataset was used for validation. The test set was not applied in training, but it was used to evaluate the generalization performance of the developed network. All of the models presented in this study were developed while using MATLAB R2018a.

### 4.1. BP ANN

There are several BP algorithms that can be applied in ANNs, such as the Powell Beale conjugate gradient, BFGS Quasi Newton, and Bayesian regularization. Among these BP algorithms, the Levenberg–Marquardt algorithm, which has been used most commonly in training networks, owing to its high speed and robustness, was adopted in this study to train the ANNs [53,54]. It has been utilized for developing predictive models for concrete properties, and its effectiveness as compared with other BP algorithms has been proven [14,15,21]. 

Before the training of the network, all of the input and target values were normalized within the range [−1, 1] while using the following equation:

(6) $$V_{norm}=2\left(\frac{V-V_{min}}{V_{max}-V_{min}}\right)-1,$$

where V and $V_{norm}$ represent the raw and normalized values, respectively. $V_{max}$ and $V_{min}$ indicate the largest and smallest values of V, respectively. Normalization of the data can improve the efficiency of learning and simplify the design procedure [39]. 

**Figure 3:**
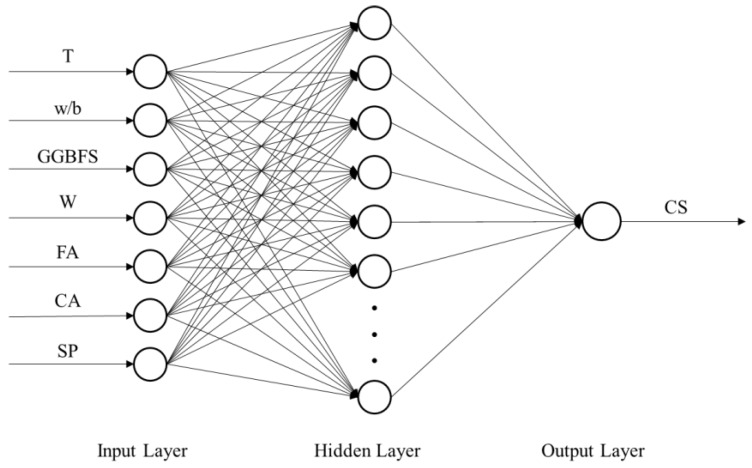
Architecture of the compressive strength (CS) prediction neural network model.

The performance of ANNs depends strongly on the network architecture and parameters, including the number of hidden layers, number of neurons in each hidden layer, and activation functions. According to various researchers, ANNs with only one hidden layer can solve almost all engineering problems [55,56,57] and generally produce excellent results [53]. Therefore, all of the ANN-based predictive models that were constructed in this study had a single hidden layer. Figure 3 shows the architecture of the CS prediction ANN models. The hyperbolic tangent function and linear function were used as the activation functions of the hidden and output neurons, respectively. 

As highlighted by several researchers, determining the number of neurons in the hidden layer (*N_h_*) is a critical task, because this number significantly affects the performance of ANNs. However, there is no theoretical rule for selecting the proper value of *N_h_*. Therefore, in this study, it was determined through trial and error. Several different ANNs were constructed with various values of *N_h_* within a reasonable range based on previously proposed empirical equations [55,58,59,60,61,62], and their performances were evaluated while using the coefficient of determination (*R*^2^) to obtain the optimal value. Table 2 presents the equations used to decide the *N_h_* range for the CS model. As shown, the *N_h_* range (2,21) was selected for the CS prediction BP ANN. The models with different *N_h_* values were each run 10 times, and the average *R*^2^ values of both the training and testing sets were computed to determine the optimal number of hidden neurons. The BP model with 15 hidden neurons exhibited the best performance; thus, a 7-15-1 architecture was applied to the BP ANN models in this study. Additional details regarding the specifications of the best BP ANN model for predicting the CS are presented later. 

### 4.2. PSO ANN

The PSO ANN represents the ANN model that was trained by the PSO algorithm, in which the positions of the particles indicate the weights and biases of the ANN. The parameters that are associated with PSO and the ANN should be selected properly to achieve the best performance of the PSO ANN. However, the parameters that lead to the minimum of the cost function are not the same in all cases, and there is no theoretical approach for identifying the optimal values. In this study, to construct a robust and accurate predictive model, the ANN parameter, i.e., the network architecture, and the PSO parameters, including the number of particles in the swarm (*Nop*) and the acceleration coefficients (*c*_1_, *c*_2_), were determined through parametric analyses. The inertia weight (*w*)—one of the PSO parameters—was taken as a random number within the range of (0, 1) [25,63]. Various values that were suggested in the previous studies were considered to find the optimal parameters, as shown in Table 3.

Each time that a network was trained, the training was stopped when the termination criteria were satisfied, i.e., the iteration number reached the limit of 2000 or the improvement in the cost function was <10^−8^ for 100 successive iterations [25]. The models with different parameters were each trained five times, and *R*^2^, as a performance measure, was calculated for the training and testing data in every run. The best model was selected according to the average values of *R*^2^ through the same method that was described in the previous section. The best result was obtained when the number of hidden neurons was 15 (as in the case of the BP ANN), the swarm size was 30, and *c*_1_ and *c*_2_ were 1.5 and 2.5, respectively. 

### 4.3. PSO-BP ANN

Hybrid algorithms combining PSO and BP have been used in ANNs to solve several engineering problems, owing to their fast convergence and global optimization capability. In the PSO-BP network model, the PSO algorithm attempted to find the near-global optimal initial points instead of random initial weights for the BP training of the ANN. The parameters that were associated with both algorithms were specified as the values determined in Section 4.1 and Section 4.2.

## 5. Evaluation of CS Prediction Models

The CS prediction ANN models trained by the BP, PSO, and PSO-BP algorithms were evaluated and compared. Each model was run 15 times with different training and testing data, and the results were evaluated with regard to the prediction accuracy, efficiency, and stability through a threefold procedure.

The four statistical indices that were employed to evaluate the performance capacity and prediction accuracy of each CS prediction model. The RMSE, mean absolute error (MAE), mean absolute percentage error (MAPE), and coefficient of determination (*R*^2^) were the main criteria that were used for performance measurement. These indices are defined as follows:

(7) $$\mathrm{MAE}=\frac{1}{n}\overset{n}{\underset{i=1}{\sum }}\left|t_{i}-o_{i}\right|$$

(8) $$\mathrm{RMSE}=\sqrt{\frac{1}{n}\overset{n}{\underset{i=1}{\sum }}{\left(t_{i}-o_{i}\right)}^{2}}$$

(9) $$\mathrm{MAPE}=\frac{1}{n}\overset{n}{\underset{i=1}{\sum }}\left|\frac{t_{i}-o_{i}}{t_{i}}\right|$$

(10) $$R^{2}={\left[\frac{\sum_{i=1}^{n}\left(o_{i}-\bar{o}\right)\left(t_{i}-\bar{t}\right)}{\sqrt{\sum_{i=1}^{n}{\left(o_{i}-\bar{o}\right)}^{2}\sum_{i=1}^{n}{\left(t_{i}-\bar{t}\right)}^{2}}}\right]}^{2}$$

where o is the predicted value of the compressive strength, t is the experimental value, n is the total number of data, $\bar{o}$ is the mean value of the predicted strength, and $\bar{t}$ is the mean value of the experimental strength. Lower values of the MAE, RMSE, and MAPE and higher values of *R*^2^ indicate a better predictability of the models.

Table 4 presents the performance indices of the best BP ANN, PSO ANN, and PSO-BP ANN models. As shown, among the developed models, the model that was trained by the hybrid algorithm had the lowest MAE, RMSE, and MAPE, as well as the highest *R*^2^, for both the training and testing datasets, which indicated that this model could predict the CS with the highest accuracy. Furthermore, the difference between the statistical performance results for the training and testing data was the smallest for the hybrid model. This result reveals the PSO-BP network model has better generalization performance than the other models. 

**Figure 4:**
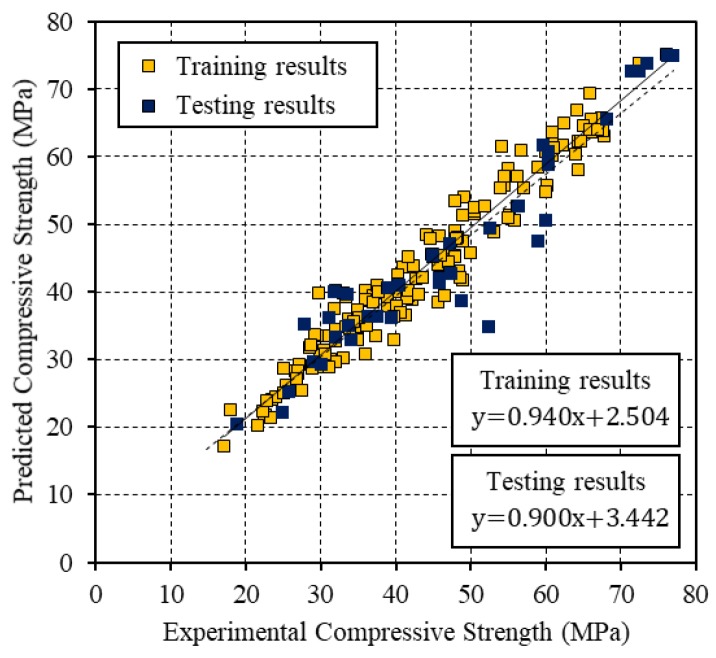
Comparison between the experimental CS and that predicted by the BP neural network.

**Figure 5:**
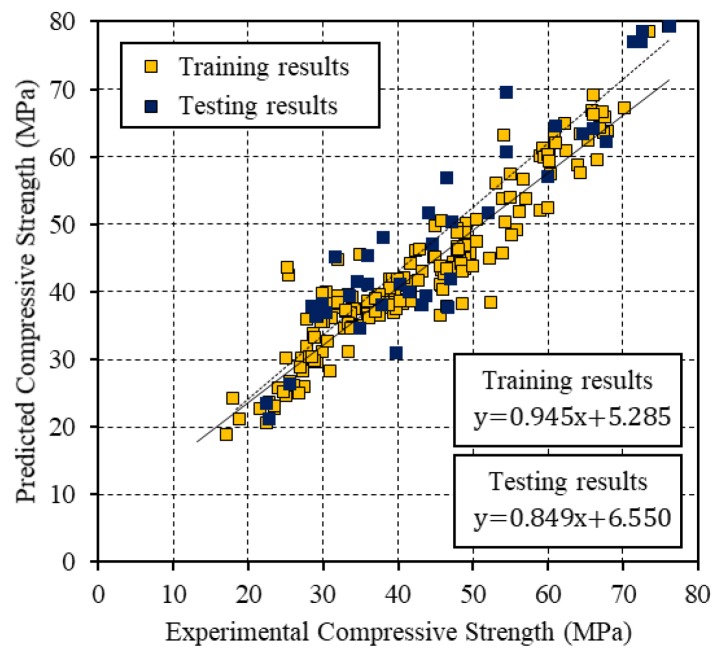
Comparison between the experimental CS and that predicted by the PSO neural network.

**Figure 6:**
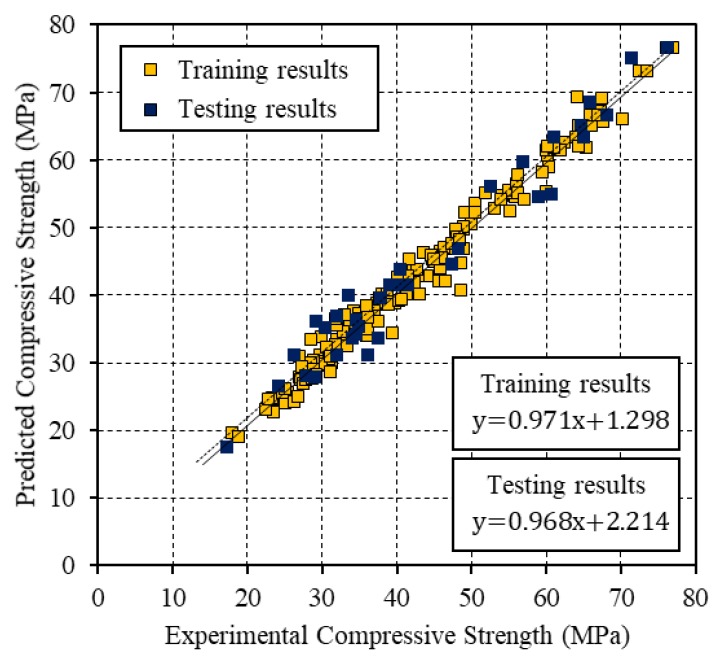
Comparison between the experimental CS and that predicted by the PSO-BP neural network.

Figure 4, Figure 5 and Figure 6 present the relationships between the experimental CS and the values that were predicted by the BP, PSO, and PSO-BP networks, respectively. The BP and PSO-BP models exhibited *R*^2^ values of >0.9 for both the training and testing datasets, which indicated that these models can provide reliable outputs with a high degree of fitness to the actual values. Thus, they are suitable for predicting the CS of GGBFS based on the mixture constituents and curing temperature. The relatively high *R*^2^ values of the proposed PSO-BP model suggest that it has the potential for estimating strength more accurately than the other models. 

**Figure 7:**
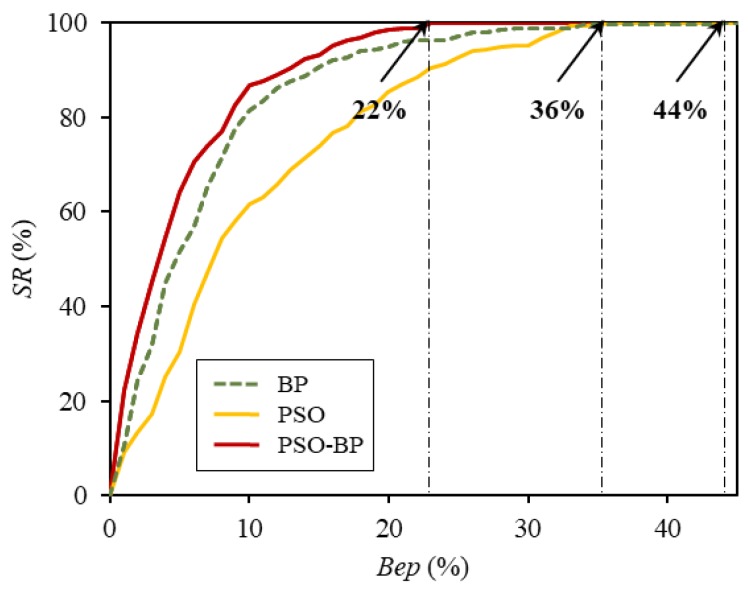
Percentage of data that have equal or smaller relative error than the specified error criterion (*SR*) for the developed models.

To perform a detailed assessment, the computational efficiency of each model was evaluated while using the *SR* [64], which is given by the following equations:

(11) $$ep_{i}=\left|\frac{t_{i}-o_{i}}{t_{i}}\right|\times 100\%,SR=\frac{N_{Bep}}{N}\times 100\%,$$

where $ep_{i}$ is the relative error and $t_{i}$ and $o_{i}$ are the measured and predicted values, respectively, of the ith data entry in the dataset. $N_{Bep}$ is the number of data entries, the relative error of which is smaller than the restrained error bound Bep (i.e., the number of entries within the area $ep_{i}<Bep$), and N is the total number of data in the considered set. The *SR* is the percentage of data that have equal or smaller relative error than the specified error criterion and it has been used for the estimation of the numerical efficiency and validity of the developed models in several studies [64]. The *SR* of each model was computed with the variation of the restrained error Bep from 0 to 100%. Figure 7 and Table 5 show the obtained results. When Bep was 5%, the *SR* for the PSO-BP ANN model was 64.1%, and those for the conventional BP ANN and the PSO ANN were 49.2% and 30.2%, respectively. These results indicate that 64.1% of the data were well-predicted by the hybrid model, with accuracy of ep<5%. As shown in Figure 7, for all values of Bep, including 5%, the *SR* of the PSO-BP network model was greater than that of the other models. Additionally, for the PSO-BP ANN, the relative error of the entire data was not greater than 22%; that is, when the restrained error Bep was 22%, the *SR* was 100%. In comparison, the prediction errors of all the data for the ANNs that were trained by the BP algorithm alone and the PSO algorithm alone were equal to or smaller than 43% and 35%, respectively. These results indicate that the hybrid prediction model has better validity and efficiency than the other models for predicting the CS of GGBFS concrete. 

Finally, to evaluate the stability of the developed models, the standard deviations of the RMSE for the models that were trained with 15 randomly selected training samples were calculated and compared. An ANN-based predictive model can give different outputs and have different performance for the same inputs, depending on the initial weight and bias values or the data-splitting method [24]. This property can cause significant problems in practical application [53,65]. Therefore, the stability of an ANN model must be validated prior to use [65,66]. In this study, as mentioned previously, each model was trained 15 times while using different combinations of training and testing sets and, then, the standard deviation (S) of the RMSE was computed while using Equation (12) to evaluate the stability of the developed models. The standard deviation indicates the sensitivity of the prediction performance of a model to the data used to train and develop it. A model with higher standard deviation is more strongly dependent on the training observations.

(12) $$\bar{X}=\frac{1}{N}\overset{N}{\underset{k=}{\sum }}X_{k},\;S=\sqrt{\frac{1}{N}\overset{N}{\underset{k=1}{\sum }}{\left(X_{k}-\bar{X}\right)}^{2}}.$$

**Figure 8:**
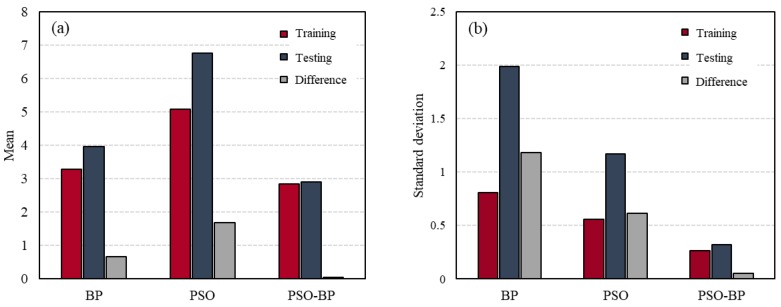
(**a**) Mean and (**b**) standard deviation of the root mean squared error (RMSE) for the developed models.

Here, N is the total number of training data and $X_{k}$ is the RMSE for the kth training set. $\bar{X}$ denotes the mean value of the RMSE for models that are trained by a specific algorithm with 15 randomly selected training samples. Figure 8 and Table 6 show the standard deviations and mean values for the BP, PSO, and PSO-BP ANN models that are based on the training and testing datasets. The BP ANN model had lower means and higher standard deviations of RMSE than PSO ANN model. These results show that the ANN models trained by BP algorithm have better prediction accuracy, but lower stability than the PSO ANN models. The standard deviations and means of the PSO-BP ANN model for both the training and testing data were smaller than those of the other models, which indicates that the model based on the hybrid algorithm was less influenced by the data splitting. Moreover, the difference between the standard deviations for the two datasets was the smallest for the PSO-BP model. As a result, it can be concluded that the PSO-BP neural network model is the most stable and accurate among the three models for estimating the CS of GGBFS concrete.

## 6. Conclusions

The ANN models were constructed to predict the CS of GGBFS concrete based on the concrete mix proportions and curing temperature while using three different learning algorithms: BP, PSO, and PSO-BP. The parameters that were associated with each algorithm or neural network were determined via a trial-and-error method, and the proposed models were trained while using 269 data divided into two sets: testing and training. The developed PSO-BP neural network model was compared with ANN models that were trained by either BP or PSO to verify its accuracy, efficiency, and stability in prediction and to prove the synergetic benefits of using the hybrid algorithms.

The PSO-BP neural network model had the lowest values of the RMSE, MAE, and MAPE, as well as the highest values of *R*^2^ for both the training and testing data, and the deviation between the results that were obtained from the training and testing data was the smallest for the PSO-BP network. These results indicate that the proposed hybrid model has the best fit for not only training data, but also unseen data. 

As shown in Table 5 and Figure 7, the hybrid model also had the highest SR for the specified error limit; i.e., its maximum relative error was smaller than those of the other two models. Additionally, when the models were trained with 15 randomly selected training samples, the PSO-BP network model exhibited the lowest standard deviation and mean values of the RMSE, which demonstrates that its prediction performance was the least affected by data division.

Several performance analyses indicated that the PSO-BP ANN model offers more accurate, reliable, and stable prediction of the CS of GGBFS concrete than the other models. That is, it has the best predictability and generalization performance among the developed models in this study. According to the results, it is obvious that using the hybrid algorithm has synergistic benefits for the performance of ANN models and the proposed hybrid PSO-BP ANN model is reliable for estimating the CS of GGBFS concrete.

## Figures and Tables

**Table 1:** Ranges of the input and output parameters in the database.

Parameters	Symbol	Unit	Category	Min	Max
Curing temperature	T	°C	Input	5	75
Water to binder ratio	w/b	%	Input	25	88.9
Water	W	kg/m^3^	Input	128	295
GGBFS to total binder ratio	GGBFS/B	%	Input	0	85
Fine aggregate	FA	kg/m^3^	Input	395	947
Coarse aggregate	CA	kg/m^3^	Input	723	1135
Superplasticizer	SP	%	Input	0	2.9
Compressive strength	CS	MPa	Output	17.2	77

**Table 2:** Empirical equations for the number of hidden neurons (*N_h_*).

Empirical Equation	Reference	
0.75*N_i_*	Neville (1986)	[58]
2*N_i_* + 1	Hecht-Nielsen (1987)	[55]
3*N_i_*	Hush (1989)	[59]
2*N_i_*	Gallant (1993)	[60]
*N_i_* + 1	Tamura (1997)	[61]
(4*N_i_*^2^ + 3)/(*N_i_*^2^ − 8)	Sheela (2013)	[62]

*N_i_* is the number of input neurons.

**Table 3:** Values of the PSO parameters considered.

Acceleration Coefficient (*c*_1_*, c*_2_)		Swarm Size (*Nop*)	Number of Hidden Neurons (*N_h_*)
*c*_1_ = 0.8, *c*_2_ = 3.2	*c*_1_ = 3.2, *c*_2_ = 0.8	10	2–21
*c*_1_ = 1.333, *c*_2_ = 2.667	*c*_1_ = 2, *c*_2_ = 1.5	20	
*c*_1_ = 1.714, *c*_2_ = 2.286	*c*_1_ = 2, *c*_2_ = 1	30	
*c*_1_ = 2, *c*_2_ = 2	*c*_1_ = 1, *c*_2_ = 2	40	
*c*_1_ = 2.286, *c*_2_ = 1.714	*c*_1_ = 1.5, *c*_2_ = 2	50	
*c*_1_ = 1.333, *c*_2_ = 2.667	*c*_1_ = 1.5, *c*_2_ = 1.5	100	

**Table 4:** Obtained statistical performance values for the developed models.

Statistical Indices	BP		PSO		PSO-BP	
	TR	TS	TR	TS	TR	TS
MAE	2.446	3.325	3.196	4.663	1.581	2.689
RMSE	3.123	5.045	4.400	5.822	2.253	3.332
MAPE	0.0595	0.0778	0.0821	0.114	0.0392	0.0644
*R* ^2^	0.943	0.906	0.884	0.861	0.971	0.961

TR and TS represent the training and testing datasets, respectively.

**Table 5:** Values of the *SR* for the developed models.

Learning Algorithm	*SR* (%)					*Bep* (%) (*SR* = 100%)
	*Bep* = 5%	*Bep* = 10%	*Bep* = 20%	*Bep* = 30%	*Bep* = 40%	
BP	49.2	81.4	94.0	98.8	99.6	44
PSO	30.2	61.7	85.5	95.1	100	36
PSO-BP	64.9	89.5	99.2	100	100	22

*Bep* represents the restrained error.

**Table 6:** Mean and standard deviation of the RMSE for the developed models.

Learning Algorithm	TR		TS	
	Mean	Standard Deviation	Mean	Standard Deviation
BP	3.185	0.828	3.959	1.989
PSO	5.079	0.557	6.767	1.170
PSO-BP	2.630	0.271	2.905	0.319

TR and TS represent the training and testing datasets, respectively.

## Appendix A

**Table A1:** The dataset used in this research.

No	T (°C)	w/b (%)	GGBFS/B (%)	W (kg/m^3^)	FA (kg/m^3^)	CA (kg/m^3^)	SP (%)	CS (MPa)	No	T (°C)	w/b (%)	GGBFS/B (%)	W (kg/m^3^)	FA (kg/m^3^)	CA (kg/m^3^)	SP (%)	CS (MPa)
1	60	25	0	163	682	882	0.8	66.2	20	20	41.7	30	156	848	933	0.95	39.01
2	60	25	40	163	647	897	0.65	65.91	21	20	44.1	50	165	835	918	0.6	38.11
3	60	25	50	163	641	899	0.7	64.13	22	20	43	50	161	840	924	0.7	41.51
4	60	25	60	163	634	901	0.75	70.32	23	20	42	50	157	845	929	0.8	40.36
5	60	25	70	163	628	903	0.7	62.27	24	20	40.9	50	153	850	934	0.95	43.69
6	60	27.5	0	163	703	909	0.77	60.34	25	20	44.1	70	165	832	915	0.5	45.79
7	60	27.5	40	163	685	911	0.6	62.48	26	20	42.8	70	160	838	922	0.6	47.27
8	60	27.5	50	163	678	913	0.63	67.78	27	20	41.4	70	155	845	929	0.75	45.76
9	60	27.5	60	163	671	916	0.7	65.39	28	20	40.1	70	150	851	936	0.9	41.76
10	60	27.5	70	163	665	918	0.68	54.5	29	20	44.1	0	165	850	916	0.65	43.14
11	60	30	0	163	721	932	0.8	60.68	30	20	43.3	70	162	850	916	0.65	45.74
12	60	30	40	163	719	919	0.63	56.85	31	20	42.5	50	159	851	918	0.7	46.63
13	60	30	50	163	712	921	0.6	64.07	32	20	41.7	30	156	852	919	0.7	48.72
14	60	30	60	163	706	924	0.68	60.24	33	20	43.1	0	168	857	888	0.608	37.83
15	60	30	70	163	699	927	0.7	50.6	34	20	42.8	20	167	847	895	0.607	38.55
16	20	44.1	0	165	841	925	0.65	41.67	35	20	42.6	30	166	841	900	0.607	38.74
17	20	44.1	30	165	837	921	0.65	33.03	36	20	42.3	50	165	828	911	0.607	40.18
18	20	43.3	30	162	841	925	0.8	36.01	37	20	41.8	70	163	811	928	0.508	38.04
19	20	42.5	30	159	845	929	0.85	37.68	38	20	28.6	0	166	757	879	1.309	67.38
39	20	28.4	20	165	742	884	1.208	68.05	59	20	49.2	30	160	863	949	0.1	29.9
40	20	28.3	30	164	725	899	1.158	67.53	60	20	49.2	30	146	863	949	0.1	30.3
41	20	27.9	50	162	707	914	0.857	67.62	61	20	49.2	30	128	863	949	0.1	30.1
42	20	27.6	70	160	690	929	0.656	66.18	62	20	49.2	40	160	863	949	0.1	32
43	10	43.1	0	168	857	888	0.608	33.05	63	20	49.2	40	146	863	949	0.1	30.4
44	10	42.8	20	167	847	895	0.607	36.03	64	20	44.5	30	160	775	923	0.1	55.3
45	10	42.3	50	165	828	911	0.607	36.13	65	20	44.5	30	137	775	923	0.1	49.1
46	10	28.6	0	166	757	879	1.309	59.42	67	20	44.5	40	160	775	923	0.1	55.9
47	10	28.4	20	165	742	884	1.208	59.55	68	20	44.5	40	137	775	923	0.1	54.4
48	10	27.9	50	162	707	914	0.857	54.23	69	20	44.5	50	160	775	923	0.15	52.6
49	15	43.1	0	168	857	888	0.608	34.32	70	20	44.5	50	137	775	923	0.15	50.5
50	15	42.8	20	167	847	895	0.607	33.78	71	20	50	30	163	836	965	1.63	39.8
51	15	42.3	50	165	828	911	0.607	33.44	72	20	50	40	163	834	964	1.63	37.4
52	15	28.6	0	166	757	879	1.309	65.03	73	20	50	50	163	834	963	1.63	35
53	15	28.4	20	165	742	884	1.208	61.07	74	20	40	30	163	764	968	2.04	48.2
54	15	27.9	50	162	707	914	0.857	61	75	20	40	40	163	763	966	2.04	45.9
55	20	47.4	0	173	873	949	0.5	52.4	76	20	40	50	163	761	964	2.04	44.1
56	20	47.4	50	173	873	949	0.5	29.7	77	45	55	0	175	853	1000	0.75	55
57	20	49.2	20	160	863	949	0.15	30.4	78	45	55	30	175	822	1014	0.6	60
58	20	49.2	20	146	863	949	0.15	31.3	79	45	55	50	175	801	1029	0.6	59
80	45	55	70	175	799	1026	0.6	50	100	60	35	70	175	694	969	0.65	29
81	45	45	0	175	817	968	0.7	49	101	75	55	0	175	853	1000	0.75	61
82	45	45	30	175	795	980	0.6	49	102	75	55	30	175	822	1014	0.6	59
83	45	45	50	175	774	995	0.55	48	103	75	55	50	175	801	1029	0.6	56
84	45	45	70	175	771	991	0.55	41	104	75	55	70	175	799	1026	0.6	48
85	45	35	0	175	741	952	0.8	39	105	75	45	0	175	817	968	0.7	48
86	45	35	30	175	718	962	0.7	36	106	75	45	30	175	795	980	0.6	45
87	45	35	50	175	698	974	0.65	35	107	75	45	50	175	774	995	0.55	49
88	45	35	70	175	694	969	0.65	32	108	75	45	70	175	771	991	0.55	37
89	60	55	0	175	853	1000	0.75	61	109	75	35	0	175	741	952	0.8	36
90	60	55	30	175	822	1014	0.6	62	110	75	35	30	175	718	962	0.7	37
91	60	55	50	175	801	1029	0.6	55	111	75	35	50	175	698	974	0.65	36
92	60	55	70	175	799	1026	0.6	43	112	75	35	70	175	694	969	0.65	31
93	60	45	0	175	817	968	0.7	49	113	20	42	0	168	783	972	0.715	46.1
94	60	45	30	175	795	980	0.6	48	114	20	42	30	168	780	968	0.463	44.9
95	60	45	50	175	774	995	0.55	48	115	20	42	50	168	778	966	0.413	44.6
96	60	45	70	175	771	991	0.55	36	116	20	37	0	168	729	981	0.817	50.5
97	60	35	0	175	741	952	0.8	40	117	20	37	30	168	726	977	0.515	56.3
98	60	35	30	175	718	962	0.7	39	118	20	37	50	168	724	974	0.455	57.1
99	60	35	50	175	698	974	0.65	32	119	20	38	0	168	672	981	1.018	60
120	20	38	30	168	668	976	0.868	64.3	140	20	85.8	17.6	219	729	1106	0	23.6
121	20	38	50	168	665	972	0.768	66.7	141	20	74.6	30	224	707	1072	0	30
122	20	27	0	168	608	965	1.52	71.4	142	20	64.1	41.6	231	677	1027	0	34
123	20	27	30	168	604	959	1.37	72.6	143	20	57.1	50	240	645	979	0	34.9
124	20	27	50	168	601	954	1.27	76.2	144	20	52.2	56.2	251	611	927	0	34.5
125	20	41	0	170	697	1035	2.9	48.3	145	20	48.3	61	261	578	877	0	31.8
126	20	41	10	170	697	1035	2.9	48.1	146	20	66.2	0	232	684	1037	0	35
127	20	41	30	170	697	1035	2.9	47.1	147	20	88.9	0	218	735	1114	0	22.6
128	20	41	50	170	697	1035	2.9	46.4	148	20	75.6	17.6	225	708	1073	0	29
129	20	56	0	168	750	1080	0	53.1	149	20	65.7	30	230	683	1036	0	36.1
130	20	56	55	168	750	1080	0	42.5	150	20	56.9	41.6	239	647	982	0	41.4
131	20	56	85	168	750	1080	0	33.5	151	20	51	50	250	609	924	0	42.3
132	20	87.6	0	219	732	1111	0	22.7	152	20	46.9	56.2	263	569	864	0	41.5
133	20	87.6	17.6	215	748	1135	0	18.1	153	20	44.2	61	279	526	799	0	37.5
134	20	87.6	30	218	731	1109	0	23.5	154	20	59.7	0	239	659	999	0	40.4
135	20	74.3	41.6	223	707	1073	0	27	155	20	80	0	224	716	1087	0	27.5
136	20	65.7	50	230	681	1033	0	27.8	156	20	67.9	17.6	231	686	1041	0	33.7
137	20	59.5	56.2	238	654	991	0	27.2	157	20	59	30	236	659	999	0	41.8
138	20	55.1	61	248	624	948	0	25.1	158	20	51.4	41.6	247	617	936	0	47.5
139	20	75	0	225	708	1075	0	28.9	159	20	46.9	50	263	570	866	0	48.4
160	20	43.4	56.2	278	525	796	0	47	180	20	41.4	70	155	845	929	0.75	45.76
161	20	40.9	61.1	295	477	723	0	42.7	181	20	40.1	70	150	851	936	0.9	41.76
162	20	50	0	185	850	952	0.5	41.5	182	20	44.1	0	165	850	916	0.65	43.14
163	20	50	10	185	850	952	0.5	40.1	183	20	43.3	30	162	850	916	0.65	45.74
164	20	50	20	185	850	952	0.5	40.6	184	20	42.5	50	159	851	918	0.7	46.63
165	20	50	30	185	850	952	0.5	39.8	185	20	41.7	70	156	852	919	0.7	48.72
166	20	50	40	185	850	952	0.5	39.4	186	20	47.9	0	163	947	870	0.7	25.5
167	20	50	50	185	850	952	0.5	37.5	187	20	47.9	60	163	939	863	0.5	23.3
168	20	50	60	185	850	952	0.42	35.2	188	20	45.3	0	163	920	880	0.7	28.2
169	20	44.1	0	165	841	925	0.65	41.67	189	20	45.3	60	163	913	873	0.55	26.6
170	20	44.1	30	165	837	921	0.65	33.03	190	20	42.9	0	163	894	890	0.7	32.9
171	20	43.3	30	162	841	925	0.8	36.01	191	20	42.9	60	163	886	882	0.5	29.2
172	20	42.5	30	159	845	929	0.85	37.68	192	20	45.3	0	163	920	880	0.7	28.5
173	20	41.7	30	156	848	933	0.95	39.01	193	20	45.3	30	163	916	876	0.6	26.2
174	20	44.1	50	165	835	918	0.6	38.11	194	20	45.3	60	163	913	873	0.55	27.1
175	20	43.0	50	161	840	984	0.7	41.51	195	20	42	0	168	783	972	0.715	46.1
176	20	42.0	50	157	845	929	0.8	40.36	196	20	42	30	168	780	968	0.463	44.9
177	20	40.9	50	153	850	934	0.95	43.69	197	20	42	50	168	778	966	0.413	44.6
178	20	44.1	70	165	832	915	0.5	45.79	198	20	37	0	168	729	981	0.817	50.5
179	20	42.8	70	160	838	922	0.6	47.27	199	20	37	30	168	726	977	0.515	56.3
200	20	37.0	50	168	724	974	0.465	57.1	220	23	50	30	168	790	995	1.05	31.1
201	20	32.0	0	168	672	981	1.018	60	221	23	50	40	168	789	994	1.05	32
202	20	32.0	30	168	668	976	0.868	64.3	222	23	50	50	168	788	993	1.16	33.5
203	20	32.0	50	168	665	972	0.767	66	223	23	45.0	30	158	760	1039	1.46	33.9
204	20	27.0	0	168	608	965	1.52	71.4	224	23	45.0	40	158	759	1038	1.47	34.5
205	20	27.0	30	168	604	959	1.37	72.6	225	23	45.0	50	158	758	1037	1.48	36.2
206	20	27	50	168	601	954	1.27	76.2	226	20	42	0	168	783	972	0.715	46
207	20	50	0	175	800	965	0.645	32	227	20	42	30	168	780	968	0.463	45
208	20	50	10	175	799	963	0.605	31.8	228	20	42	50	168	778	966	0.413	44.7
209	20	50	30	175	797	960	0.585	29.8	229	20	37	0	168	729	981	0.817	50.5
210	20	50	50	175	794	957	0.545	28.5	230	20	37.0	30	168	726	977	0.515	52
211	20	30	0	165	717	924	0.65	56.3	231	20	37.0	50	168	724	974	0.465	54
212	20	30	65	165	705	909	0.3	54.5	232	20	32.0	0	168	672	981	1.018	60.3
213	20	27	65	163	689	887	0.3	61.1	233	20	32.0	30	168	668	976	0.868	64.7
214	23	55	0	179	819	952	0.73	27.8	234	20	32.0	50	168	665	972	0.767	67
215	23	50	0	168	793	999	1.04	28.7	235	20	27.0	0	168	608	965	1.52	72.4
216	23	45	0	158	764	1043	1.46	30.7	236	20	27.0	30	168	604	959	1.37	73.5
217	23	55	30	179	817	949	0.84	28.9	237	20	27.0	50	168	601	954	1.27	77
218	23	55	40	179	815	947	1.05	29.3	238	5	60.0	0	217.2	399	912	0.65	24.8
219	23	55	50	179	814	946	1.05	31.2	239	5	60.0	10	217.2	397	910	0.65	22.4
240	5	60.0	30	217.2	396	908	0.65	18.8	260	20	60.0	20	172	877	996	0.5	52.2
241	5	60.0	50	217.2	395	906	0.65	17.2	261	20	57.0	20	175	843	997	0.55	46.3
242	20	60.0	0	217.2	399	912	0.65	26.4	262	20	50.0	20	176	811	998	0.5	39
243	20	60.0	10	217.2	397	910	0.65	24.1	263	20	45.0	20	181	769	985	0.5	37.2
244	20	60.0	30	217.2	396	908	0.65	22.9	264	20	40.0	20	187	721	962	0.5	31.6
245	20	60.0	50	217.2	395	906	0.65	21.7	265	20	60.0	30	172	877	996	0.5	49
246	35	60.0	0	217.2	399	912	0.65	27.4	266	20	55.0	30	175	843	997	0.5	43.4
247	35	60.0	10	217.2	397	910	0.65	25.7	267	20	50.0	30	176	811	998	0.5	40.8
248	35	60.0	30	217.2	396	908	0.65	26.8	268	20	45.0	30	181	769	985	0.5	39.3
249	35	60.0	50	217.2	395	906	0.65	25.1	269	20	40.0	30	187	721	962	0.5	32.1
250	20	60.0	0	172	877	996	0.5	49.7									
251	20	55.0	0	175	843	997	0.5	43.3									
252	20	50.0	0	176	811	998	0.5	39.7									
253	20	45.0	0	181	769	985	0.5	37.9									
254	20	40.0	0	187	721	962	0.48	25.5									
255	20	60.0	10	172	877	996	0.5	54									
256	20	55.0	10	175	843	997	0.5	46.5									
257	20	60.0	10	176	811	998	0.5	43.3									
258	20	45.0	10	181	769	985	0.5	33.5									
259	20	40.0	10	187	721	962	0.5	25.2									
